# Evaluating the Effectiveness of the Driving Resumption Assessment in Brain Disorders: Insights From a Retrospective Observational Study

**DOI:** 10.7759/cureus.65304

**Published:** 2024-07-24

**Authors:** Yuzo Tsuda, Ryo Yoshikawa, Atsuko Matsuda, Yasumitsu Fujii, Yoshimichi Kobayashi, Risa Harada, Yoshiaki Saji, Yoshitada Sakai

**Affiliations:** 1 Department of Rehabilitation Medicine, Ishikawa Hospital, Himeji, JPN; 2 Department of Physical Medicine and Rehabilitation, Kobe University Hospital, Kobe, JPN; 3 Division of Rehabilitation Medicine, Kobe University Graduate School of Medicine, Kobe, JPN; 4 Department of Surgery, Ishikawa Hospital, Himeji, JPN

**Keywords:** rehabilitation, driving resumption assessment, neuropsychological assessment, brain disorder, driving ability

## Abstract

Objectives

For patients with brain disorders, regaining the ability to drive is crucial to their reintegration into society. Despite the existence of numerous assessment methods for determining the ability to resume driving, the most effective approach remains unclear. This study evaluated patients with brain disorders who had received support for driving resumption. We examined the factors influencing the acquisition of driving ability in this specific population.

Methods

This retrospective observational study was conducted from July 2019 to March 2022. Initially, a desk-based assessment was conducted using neuropsychological tests. Successful candidates subsequently underwent an on-road assessment at an affiliated driving school. Patients who passed both assessments were granted permission to resume driving. The participants were categorized into pass and fail groups based on their assessments, and a comparative analysis was conducted. Age, sex, type of brain disorder, functional independence measures (FIMs), assessments of higher cognitive skills, and physical function test results were evaluated.

Results

Forty-five patients (average age: 62±13 years) underwent evaluation. Logistic regression analysis for the desk-based assessment identified the Rey-Osterrieth complex figure test (ROCFT) (three-minute delayed recall) as the most influential factor (cutoff value: 21.5 points; sensitivity: 65%; specificity, 72.7%). In the on-road assessment, the 10-m walking test was significantly faster in the passing group than in the failing group (p<0.005).

Conclusions

We demonstrated that the ROCFT (three-minute delayed recall) was the most effective neuropsychological assessment tool for evaluating driving resumption. The assessment of walking speed may also be able to predict the resumption of driving in patients with brain disorders.

## Introduction

Automobile accidents involving patients with brain disorders occur frequently, and there is ongoing debate regarding the adequacy of tests determining their driving abilities. For patients with brain disorders, driving a car is an important means of reintegrating into society [1,2]. For them, the ability to drive is highly beneficial for maintaining activities of daily living (ADL) and quality of life (QOL). Therefore, in recent years, support for the resumption of driving has gained attention in rehabilitation medicine [3-6]. In the United States, higher cognitive function screening measures are recommended to assess driving ability [7]. However, the best method of assessing the driving abilities of patients with brain disorders has yet to be determined [6,8]. In Japan, there are no unified or clear criteria, and effective assessments have not yet been established. Individual medical institutions in Japan, including our hospital, develop their own criteria. Doctors make decisions regarding the resumption of driving using different assessments at each hospital. As the Japanese population ages, the number of patients with brain disorders is increasing. Therefore, it is important to assess the driving ability of these patients accurately. In this study, we evaluated patients for whom we provided support for driving resumption and examined the factors influencing the acquisition of driving abilities. The purpose of this study is to examine the higher cognitive skills and physical functions required for patients with brain disorders to resume driving. We hypothesized that attention function, visual-spatial cognition, and physical functions such as walking ability are important for resuming driving.

## Materials and methods

Ethics

This study complied with the principles of the Declaration of Helsinki for Human Research. The ethics committee of our hospital approved this study (no. 2022-1), and informed consent was obtained from the patients or their legal guardians.

Study design and patients

This retrospective observational study was conducted between July 2019 and March 2022. Patients with brain disorders who were hospitalized at our hospital and underwent assessments for the resumption of driving were recruited for this study. This study focused on patients with non-traumatic brain disorders. We excluded the patients exhibiting clinically evident severe paralysis or higher brain dysfunction.

Desk-based assessment and on-road assessment

At our hospital, when patients express a desire to resume driving, a desk-based assessment is conducted, involving several assessments of higher cognitive skills, such as the Mini-Mental State Examination (MMSE), Kohs block-design test (Kohs), Trail Making Test (TMT) Parts A and B, Clinical Assessment for Attention Test (CAT), Rey-Osterrieth Complex Figure Test (ROCFT), Behavioural Assessment of the Dysexecutive Syndrome (BADS), Wechsler Adult Intelligence Scale - Fourth Edition (WAIS-IV), and Cognitive-Related Behavioral Assessment (CBA). The attending physician comprehensively evaluates the results of these assessments to determine the overall outcome of the desk-based assessment. If the desk-based assessment is successful, a practical on-road driving evaluation is then conducted at an affiliated driving school. The criteria for passing the on-road assessments were based on the responses from driving instructors at the driving school. Finally, patients who pass both the desk-based and on-road assessments are granted permission by the attending physician to resume driving.

Evaluated factors

We evaluated factors including age, sex, type of brain disorder, functional independence measures (FIM), several assessments of higher-order cognitive skills (MMSE, Kohs, TMT, CAT, ROCFT, BADS, WAIS-IV, CBA), and physical function tests, including the Fugl-Meyer Assessment (FMA), Brunnstrom Stage (BRS), Action Research Arm Test (ARAT), Functional Ambulation Categories (FAC), 10-m walking test, Postural Assessment Scale for Stroke (PASS), and the Scale for the Assessment and Rating of Ataxia (SARA). We divided the participants into two groups, the pass and fail groups, based on their performance in the desk-based and on-road assessments, and conducted between-group comparisons.

Statistical analysis

First, the desk-based assessment results were used to classify patients into pass/fail groups, and the results of each test were compared using the Mann-Whitney U test. Logistic regression analysis with the forward-backward stepwise selection method was performed to examine the passing or failing of the desk-based assessment by age, sex, hemisphere of damage (left or right), and factors that showed significant differences as independent factors. The on-road assessment results were subsequently used to classify patients who passed the desk-based assessment into pass/fail groups, and the results of each test were also compared using the Mann-Whitney U test after confirming non-normal distribution using the Shapiro-Wilk test. All statistical analyses were performed using Statistical Product and Service Solutions (SPSS, version 26.0; IBM SPSS Statistics for Windows, Armonk, NY).

## Results

The 45 patients who were included in the study consisted of 35 men and 10 women. The average age is 62.4±1.95 years, with an average duration from onset to examination of 23.4±2.93 days. The average upper limb BRS is 5.27±0.20, hand BRS is 5.24±0.21, and lower limb BRS is 5.27±0.19 (Table 1).

**Table 1:** Characteristics of the patients BRS: Brunnstrom Stage

Average	Patients (n=45)
Age	62.4±1.95 (years)
Duration from onset to examination	23.4±2.93 (days)
Upper limb BRS	5.27±0.20
Hand BRS	5.24±0.21
Lower limb BRS	5.27±0.19

**Figure 1:**
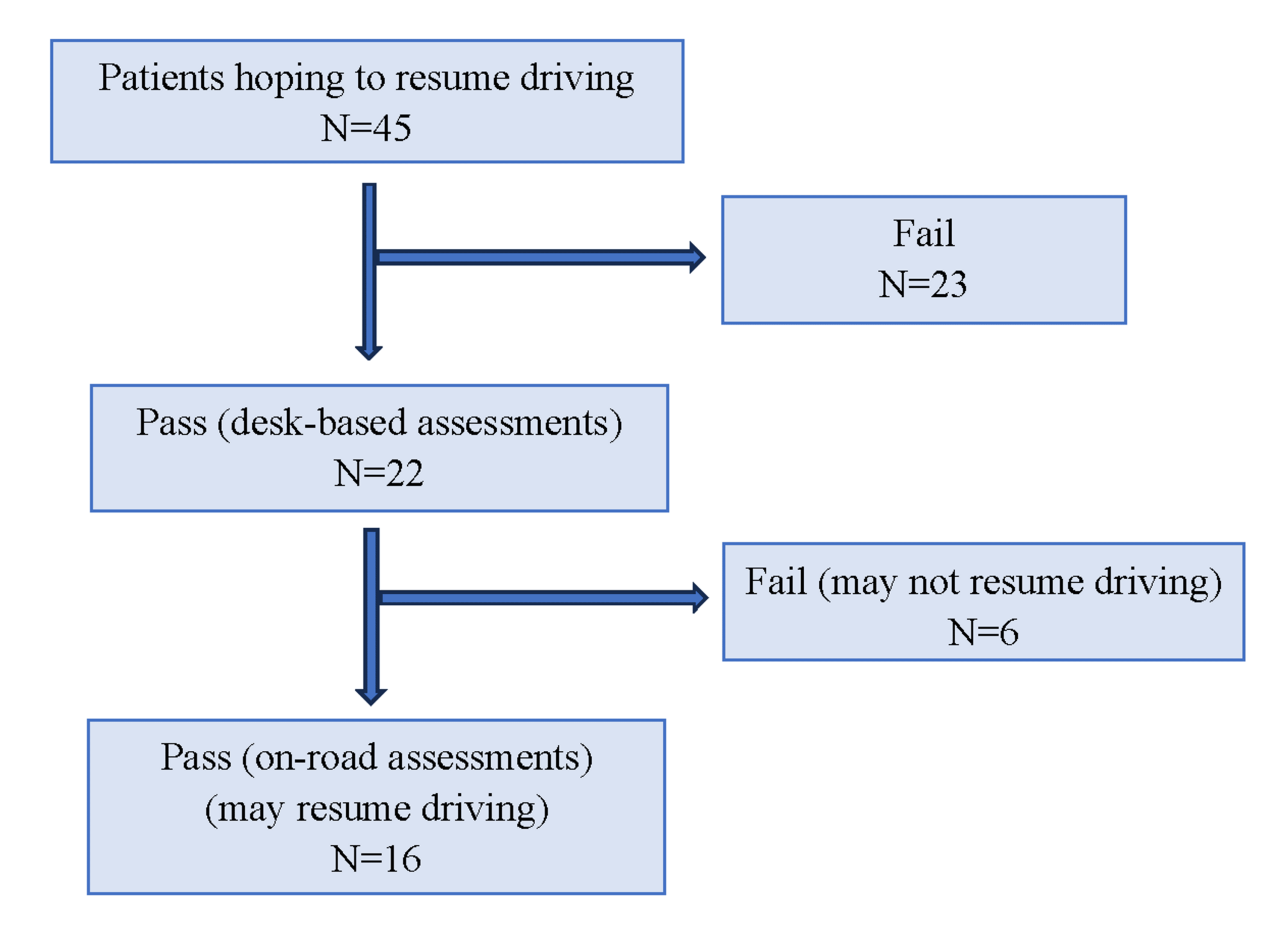
The examination results for both the desk-based and on-road assessments

The underlying causes of brain disorders were cerebral infarction in 30 patients, cerebral hemorrhage in 11, and other causes in four (meningioma, brain abscess, hypoxic brain injury, and normal pressure hydrocephalus). As shown in Figure 1, desk-based assessments were conducted for the 45 individuals who expressed a desire to resume driving, and on-road assessments were conducted for the 22 individuals who passed the desk-based assessment. Sixteen patients passed the on-road assessment and were permitted to resume driving, while six failed.

Assessment outcomes

Desk-Based Assessment

In the comparison between the pass and fail groups in the desk-based assessment, significant differences were observed in the Kohs (p=0.046), TMT Part A (p=0.008), and among the items "△" time (p=0.026), "mark" time (p=0.001), "3" accuracy rate (p=0.043), and "ka" correct response rate (p=0.019) within the visual cancellation task in the CAT, as well as in the ROCFT (three-minute delayed recall) (p=0.018) (Table 2).

**Table 2:** Comparison of the pass and fail groups in the desk-based assessment

	Pass group (n=22)	Fail group (n=23)	p-value
Kohs block-design test	97.0±19.2	84.0±23.4	0.046
Trail Making Test-Part A	42.5±14.5	61.6±28.3	0.008
Visual cancellation task ”△” time	52.6±12.2	85.0±96.2	0.026
Visual cancellation task ”mark” time	59.4±11.0	87.1±44.3	0.001
Visual cancellation task ”3” accuracy rate	100±0	99.7±0.8	0.043
Visual cancellation task ”ka” correct response rate	98.4±2.0	94.8±5.8	0.019
Rey–Osterrieth Complex Figure Test (3-min delayed recall)	23.5±7.6	17.2±8.5	0.018

However, no significant differences were observed in the age or physical function assessments (Table 3).

**Table 3:** Comparison of the pass and fail groups in the desk-based assessment

	Pass group (n=22)	Fail group (n=23)	p-value
Age	62.1±12.8	62.7±13.8	0.847
Functional Independence Measures (motor)	87.4±4.5	82.5±10.6	0.062
Functional Independence Measures (cognition)	32.6±3.1	32.6±2.9	0.913
Fugl–Meyer Assessment (upper)	61.6±5.4	60.6±6.1	0.612
Fugl–Meyer Assessment (lower)	33.4±1.1	32.3±2.1	0.094
Action Research Arm Test	54.5±7.4	50.6±9.3	0.432
Functional Ambulation Categories	4.5±0.8	3.9±1.1	0.274
10-meter walking (time)	8.1±3.9	12.0±10.0	0.156
Postural Assessment Scale for Stroke	33.7±5.9	32.9±3.9	0.314
Scale for the Assessment and Rating of Ataxia	4.8±4.8	6.3±5.6	0.914

Logistic regression analysis examining the presence or absence of passing the desk-based assessment showed a significant difference in the ROCFT (three-minute delayed recall) (Table 4).

**Table 4:** Logistic regression analysis examining the presence or absence of passing the desk-based assessment showed a significant difference in the Rey–Osterrieth complex figure test (three-minute delayed recall) Dependent variable: passing the desk-based assessment; independent variables: age, sex, hemisphere of damage (left or right), and factors that showed significant differences

	Odds ratio	p-value	95％CI	
			Lower	Upper
Rey–Osterrieth Complex Figure Test (3-min delayed recall)	1.153	0.022	1.021	1.301

**Figure 2:**
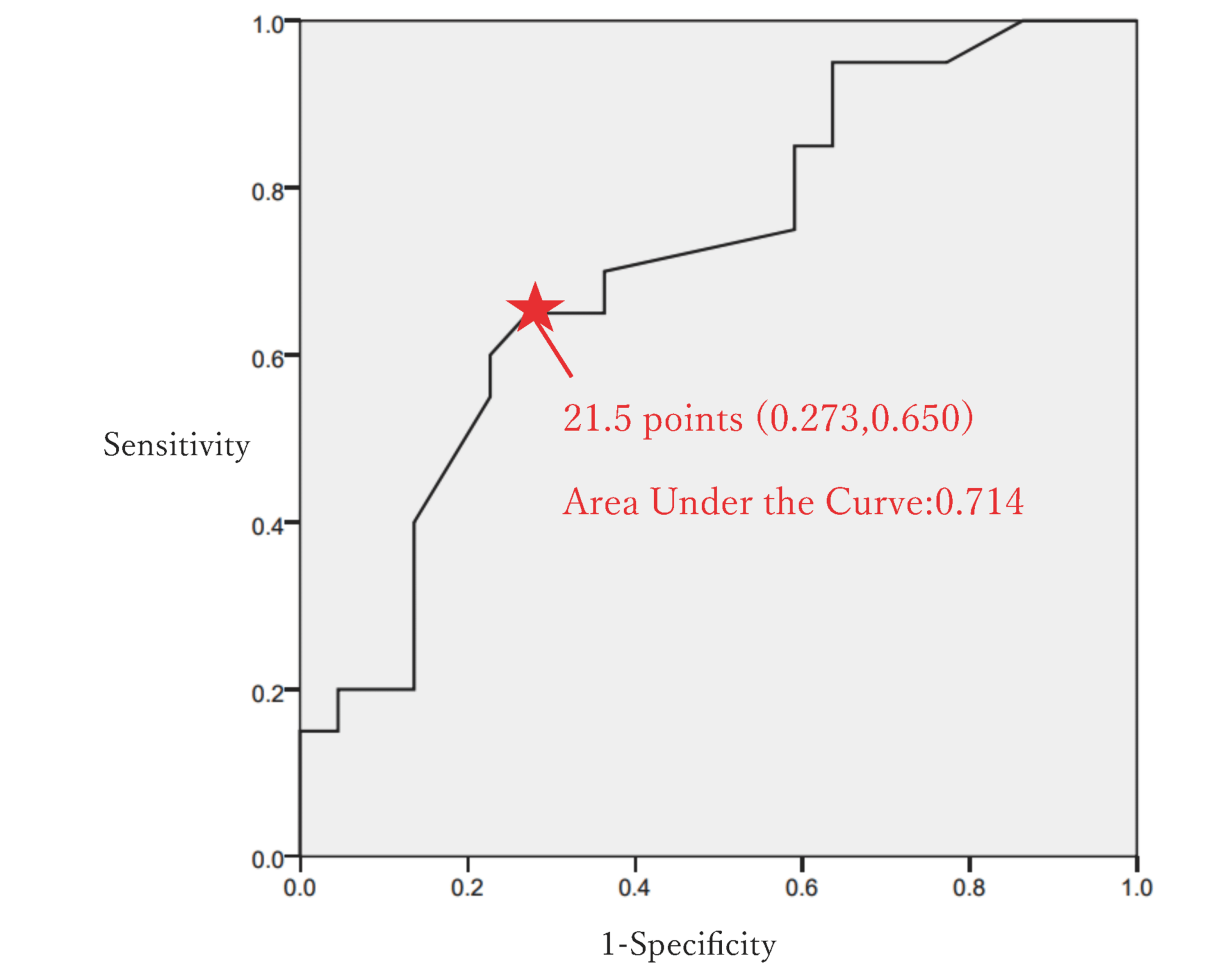
Cutoff value determination for the Rey–Osterrieth complex figure test in the desk-based assessment A receiver operating characteristic curve analysis was conducted to determine the cutoff value of the Rey–Osterrieth complex figure test (three-minute delayed recall) for the pass group in the desk-based assessment. The cutoff value was determined to be 21.5/36 points (area under the curve: 0.714, sensitivity: 65%, specificity: 72.7%).

Furthermore, receiver operating characteristic (ROC) curve analysis was conducted to determine the cutoff value of the ROCFT (three-minute delayed recall) for the pass group. The cutoff value was determined to be 21.5/36 points (AUC: 0.714; sensitivity: 65%; specificity: 72.7%) (Figure 2).

On-Road Assessment

In the comparison between the pass (driving resumption group) and fail groups (driving non-resumption group) in the on-road assessment, no significant differences were observed in the neuropsychological assessments (Table 5).

**Table 5:** Comparison of the pass and fail groups in the on-road assessment

	Pass group (n=16)	Fail group (n=6)	p-value
Mini Mental State Examination-J	29.4±1.1	29.3±0.8	0.693
Kohs block-design test	100.9±16.1	86.5±24.4	0.154
Trail Making Test-Part A	39.3±12.9	51.0±16.0	0.115
Trail Making Test-Part B	69.3±29.0	91.8±44.3	0.231
Visual cancellation task “△” time	52.0±13.0	54.2±10.3	0.747
Visual cancellation task “△” accuracy rate	99.9±0.5	100	0.858
Visual cancellation task “mark” time	59.44±11.9	59.2±8.9	0.971
Visual cancellation task “mark” correct response rate	99.9±0.5	99±1.7	0.329
Visual cancellation task “mark” accuracy rate	99.9±0.5	100	0.858
Visual cancellation task “3” time	101.9±23.2	115.5±28.1	0.407
Visual cancellation task “3” correct response rate	99.3±1.5	98.8±0.8	0.7
Visual cancellation task “3” accuracy rate	100	100	1
Visual cancellation task “ka” time	127.0±29.8	134.5±25.0	0.329
Visual cancellation task “ka” correct response rate	98.6±2.0	97.8±2.0	0.449
Visual cancellation task “ka” accuracy rate	99.3±2.8	100	0.858
Rey–Osterrieth Complex Figure Test (copy)	34.3±1.5	34.8±2.2	0.445
Rey–Osterrieth Complex Figure Test (3-min delayed recall)	23.9±7.4	22.2±8.9	0.612
Behavioural Assessment of the Dysexecutive Syndrome (zoo map)	2.6±1.5	2.8±1.6	0.905
Wechsler Adult Intelligence Scale - Fourth Edition (puzzle task)	11.1±4.1	10.6±3.4	0.842
Cognitive-Related Behavioral Assessment	28.4±1.5	27.8±1.0	0.327

However, a significant difference was observed in the 10-m walking test in the age and physical function assessments (Table 6).

**Table 6:** Comparison of the pass and fail groups in the on-road assessment

	Pass group(n=16)	Fail group(n=6)	p-value
Age	60.2±13.7	67.3±9.4	0.294
Functional Independence Measures (motor)	88.2±3.9	84.8±5.9	0.398
Functional Independence Measures (cognition)	33.0±2.9	31.2±3.6	0.398
Fugl–Meyer Assessment (upper)	61.4±5.5	65	0.75
Fugl–Meyer Assessment (lower)	33.4±1.2	34±0	0.5
Action Research Arm Test	54.4±7.8	56	0.5
Functional Ambulation Categories	4.7±0.5	3	0.25
10-meter walking (time)	6.6±2.0	13.6±4.7	0.005
Postural Assessment Scale for Stroke	35.6±1.1	29.3±10.7	0.267
Scale for the Assessment and Rating of Ataxia	6.0±5.0	1	0.5

## Discussion

In this study, we examined the test items that affect driving resumption in individuals with brain disorders. Neuropsychological assessments in the desk-based assessment, such as the Kohs, TMT Part A, visual cancellation task in CAT, and ROCFT, may have potential usefulness, with particular emphasis on the ROCFT (three-minute delayed recall). The factor influencing the resumption of driving in patients who passed the desk assessment was the 10-m walking time, suggesting the importance of lower limb function and gait ability as predictive factors for driving resumption.

Driving is a complex activity that requires various high-level cognitive skills. According to the United States guidelines, the cognitive skills used in driving are short- and long-term memory, visuospatial cognition function (including visual perception and visual processing and visual search and visuospatial skills), selective and divided attention, executive skills, language, and vigilance [7]. In driving, attention and visual-spatial cognition are essential abilities. Tests such as the TMT, CAT, and ROCFT are considered important assessments that can evaluate these necessary skills for driving. However, there is currently no established neuropsychological assessment that is effective in predicting the ability to resume driving, and there are no unified criteria between facilities. The TMT and ROCFT have previously been reported to be useful screening tools for evaluating driving ability [4]. Combining assessments that focus on attention and visuospatial cognitive function could be important. In this study, we also found that the TMT and ROCFT were useful for measuring attention and visuospatial cognitive functions, respectively.

When driving a car, attention functions such as the ability to "maintain a safe following distance," "pay attention to signals and road signs," "be aware of pedestrians and oncoming vehicles," and "switch attention in response to changing situations" are essential. TMT is reportedly both convenient and useful in the assessment of attention function [3,9,10]. Furthermore, in stroke patients, driving suitability can be predicted with 86% accuracy using three assessments: the visual cancellation task test, ROCFT, and on-road assessment [11]. The reference values for the TMT show significant variations among facilities, and there is no consensus on whether Part A or B is more useful. However, in our study, both TMT Part A and the CAT visual cancellation task were found to be useful in the desk-based assessment. These tests are considered valuable due to their simplicity and relatively short administration times.

When driving a car, visuospatial cognitive functions such as the ability to "drive within lanes accurately," "maintain a safe following distance," and "assess the speed and direction of other vehicles" are essential. The ROCFT is useful for assessing visuospatial cognitive functions [4,9,12]. Combined neuropsychological assessments, including the ROCFT and WAIS digit symbol task, classify the resumption of driving with an accuracy rate of 94.4 [13], and one report indicates that ROCFT scores correlate with the passing of the on-road assessment [6]. In addition, logistic regression analysis has identified the ROCFT as an independent predictor of on-road assessments of patients with brain disorders [14]. In our study, logistic regression analysis demonstrated the usefulness of the ROCFT. Furthermore, ROCFT scores are less affected by aphasia. Like the TMT, the ROCFT is simple and suitable for screening assessments. However, there is currently no established cutoff value for the ROCFT in predicting driving ability. In this study, the ROCFT was considered more crucial as a neuropsychological assessment for driving resumption, and a cutoff value of 21.5 points was identified.

Regarding the relationship between physical function and driving, in patients with brain disorders, individuals with higher scores on the motor items of the FIM can resume driving [15]. In patients who passed the desk-based assessment in this study, the time taken for the 10-m walking test was identified as a factor influencing the resumption of driving. This suggests that not only higher cognitive function but also motor functions, such as lower limb function and walking ability could be predictive factors for driving resumption.

This study had several limitations. First, this was a retrospective study. We are constrained by the tests that were already available at the time of data collection. This limitation can affect the comprehensiveness and relevance of the neuropsychological assessments used in our study. Further prospective studies are necessary to confirm the cutoff values in the future. Second, the sample size was small, leading to potential bias in patient backgrounds, including the causes of brain disorders. Larger-scale clinical studies are warranted in the future. Third, since our hospital did not have a driving simulator, it was not available for use in driving resumption assessments. Finally, it has not been confirmed whether patients who resumed driving could continue driving without encountering problems. Therefore, long-term follow-up is required.

## Conclusions

In this study, we examined the factors influencing the resumption of driving in patients with brain disorders. Among the neuropsychological assessments for evaluating the resumption of driving, the ROCFT score may have significant potential. As a passing criterion for the desk-based assessment, the cutoff value for the ROCFT (three-minute delayed recall) was determined to be 21.5 points. Furthermore, in addition to higher cognitive function, motor functions such as lower limb function and walking ability could emerge as potential predictive factors for driving resumption. Large-scale clinical studies with long-term follow-up are warranted in the future.
